# Chronic non-communicable disease as a new epidemic in Africa: focus on The Gambia

**DOI:** 10.11604/pamj.2013.14.87.1899

**Published:** 2013-03-05

**Authors:** Semeeh A Omoleke

**Affiliations:** 1 Medical Research Council (UK) Unit, Fajara, P.O. Box 273, Banjul, The Gambia

**Keywords:** Chronic non-communicable diseases (NCDs), Africa, The Gambia, morbidity, mortality, hospital admission, epidemiology, health services, surveillance, government

## Abstract

**Introduction:**

Recent epidemiological data suggest increasing burden of NCDs in many African countries but these diseases have not been given adequate attention due to the overwhelming burden of infectious diseases. There are no recent reports or studies on NCDs or related issues in The Gambia, consequently, this report intends to stimulate further epidemiological studies and also policy initiatives to forestall an epidemic.

**Methods:**

Routine data on morbidity (in and out-patients), hospitalisation and mortality due to NCDs from health facilities in The Gambia between 2008 and 2011 were used. Other relevant data from multiple sources were also used.

**Results:**

There is an increasing trend in the morbidity, hospitalisation and mortality due to NCDs in the Gambia between 2008 and 2011; 19.8%, 9.9% and 23.4% increments respectively. There is evidence of gender differences in these variables; more males suffer higher mortality from NCDs than females (p < 0.001). Furthermore, there is dearth of highly skilled health workforce as well as poor health infrastructures in The Gambia.

**Conclusion:**

NCDs are becoming a public health challenge and the capacity to respond to NCDs in most African countries, particularly, The Gambia is very weak. There is need for a population-based study to accurately quantify the burden and their risk factors as a first step towards policy formulation and effective implementation. Furthermore, there is dire need for increased investments on health workforce as well as medical products and technologies towards addressing the consequences of this emerging epidemic.

## Introduction

African countries are undergoing varying degree of human development, and this is being accompanied by nutritional, demographic and epidemiological transition. The traditional public health challenge in Africa has been the scourge of infectious disease, otherwise called communicable diseases such as HIV/AIDS, Tuberculosis, Malaria, Pneumonia and Diarrhoea. Generally, Africa is still besieged with this (infectious diseases) public health challenge, even though the severity of the burden varies across the continent. However, in the last three decades, there has been rapid increase in the prevalence of chronic non-communicable diseases (NCDs) such as coronary heart disease, hypertension, stroke, diabetes, asthma, chronic hepatic diseases and chronic renal diseases [1, 2]. This has resulted into double burden of diseases which is potentially overwhelming to the poorly financed health services in Africa [3–6]. Non-communicable diseases (NCDs) tend to be chronic, usually affecting adults who are economically productive and also bear enormous social responsibilities [2, 7].

Africa and most countries in Asia and Latin America bear appreciable proportion of the global burden of NCDs. NCDs kill more than 36million people annually with 80% of these occurring in low and middle income countries [2], though this has been largely overlooked due to the overwhelming burden of infectious diseases, notably HIV/AIDS, TB and Malaria. Cardiovascular and respiratory disorders, cancer and diabetes account for about 80% of all death due to NCDs [2]. Recent WHO report projects that NCDs will be the leading cause of deaths globally, exceeding communicable, maternal, perinatal and nutritional disease by 2030 [2].

Increasing trends in the burden of NCDs in low and middle income countries (LMIC) has been linked to increasing levels of the risk factors such as smoking/tobacco consumption, reduced physical activity levels, increasing life expectancy, nutritional transition from traditional to western diet and increased alcohol consumption [2]. These risk factors have been increasing at both individual and population levels. These risk factors are closely connected to westernisation or adoption of western culture and values as defined by materialism, consumerism, individualism [8]; urbanisation and economism [9]; globalisation and trade liberalisation (including the food market) [10].

There are few studies that provide data on NCDs from Africa and these are mainly from South Africa [11]. In the same vein, research efforts in The Gambia have been largely focused on infectious diseases. Specifically, no recent study evaluated NCDs and their associated risk factors in The Gambia. The only study (conducted in 1997) that investigated the prevalence of hypertension and related diseases was based on 1% sample of adult population in The Gambia [6]. The national prevalence of hypertension was found to be 24.2% [6]. The prevalence of obesity and undernutrition and cardiovascular risk factors were later investigated by the same group [12]. A recent World Health Organisation (WHO) report predicts that about the 61,000 persons; about 4% of Gambian population could be diabetic by 2030 [13]. Consequently, this study aims at stimulating further empirical (epidemiologic) studies and policy initiatives by reporting the morbidity and mortality trends of NCDs in The Gambia

## Methods

### Data

Data for this study were obtained from the Disease Control Unit of the Ministry of Health and Social Welfare, The Gambia. These were collected routinely from peripheral health centres and hospitals (including private facilities) across the country and this system has been in place since 2008, even though there are systemic challenges facing surveillance for NCDs in The Gambia, which is similar to what is obtainable in many African countries [14]. Specific data on health facilities admissions, morbidities (new in and out-patients cases with or without complications) and mortalities were obtained on cardiovascular diseases, hepatic and renal diseases, cancers and asthma for a period between 2008 and 2011. Diagnoses of these health conditions were made in most cases by doctors working at the secondary and tertiary healthcare levels. At the primary health care level, there are algorithms being used for diagnosis by Senior Community Health Nurses for some conditions and referrals were made to higher level in cases where diagnoses cannot be made. The doctors hospitalise patients and ascertain cases of mortality as required.

The Health Management Information System Unit centrally coordinates and manages health data including that of chronic non-communicable diseases in The Gambia. The software application being used for the database is DHIS version2. Data entry by data entry clerks mainly takes place at the Regional Health Level with some contributions at the peripheral levels as some major health facilities now have data entry facilities. Data were verified manually before entry from the source documents and also after data entry. Verifications were done by data entry supervisors. There are restrictions to the database as only data management team has access to it. Each patient has a unique identifier, and other mechanisms are in place to prevent duplication. The database also has protected data field that improves the data quality. There are other consistency checks and validation rules that have been put in place. As much as possible GCP, compliance was observed. The target set for timeliness and completeness for reporting from the peripheral levels was 80% which has been met and surpassed for the period being studied.

### Analysis

Proportion were obtained and test of significance were done using Epi 6 statistical package to check whether there are statistically significant differences in mortality, morbidity and hospital admission over the years studied. This study also examined gender differences in the occurrence of NCDs in The Gambia based on the available data. However, other forms of NCDs such as neuropsychiatric conditions, haemoglobinopathies and chronic lung diseases (except asthma) were excluded in the analysis.

These data were reinforced with the World Health Statistics for other useful health, demographic and socio-economic data relevant to this work. The demographic and socio-economic variables are strong determinants of health [15, 16]. Furthermore, evidence suggests a correlation between these social factors and the risk factors and prevalence levels of NCDs [2, 15].

## Results

From Table 1, it can be seen that The Gambia has increased in population over the last 10years from 1.384million to 1.705million, though the annual growth rate has slightly slowed down from 3.8% to 3.0%. There has been rapid and significant rural-urban migration; from 49% in 2000 to 57% in 2009. Fertility rate is high, though has reduced slightly in about a decade (5.6 to 5.0/woman). The Gambia has a low (45%) literacy level. Though still low, the GNI per capita has risen from $920 to $1,330 and there is high percentage of people living below the poverty level Table 2 shows increases in the three variables over a 4-year period, especially morbidity and mortality reported. Further analyses revealed that the percentage increase in total morbidity due to NCDs reported over a 4-year period is approximately 19.8% while increase in hospital admissions due to NCDs reported during the same period is approximately 9.9%. Mortality due to NCDs also rose by 23.4% between 2008 and 2011.

**Table 1:** Demographic and socio-economic data

Variables	2000	2009
Population (in millions)	1.384**	1.705
Annual Growth Rate (%)	3.8	3.0
Urban Residents (%)	49	57
Total Fertility Rate (/woman)	5.6	5.0
Adult Literacy Rate (%)	-	45
GNI per Capita Income ($)	920	1,330
Population (%) Living on <$1/day (2000-2008)	34.3	34.3

Sources: World Health Statistics, 2011

** Others: - Central Statistics Department, the Gambia

**Table 2:** Routine Hospital-Based Data for NCDs in The Gambia

2008	Cardiovascular Diseases	Diabetes	Asthma	Others	Total
Morbidity	47,258	2,153	8,934	422	58,767
Admission	1,454	182	432	135	2,203
Mortality	118	11	13	25	167
**2010**					
Morbidity	54225	3115	10174	323	67837
Admission	1458	176	450	128	2212
Mortality	72	18	18	25	133
**2011**					
Morbidity	57,208	3,232	9,621	322	70,383
Admission	1,532	203	501	184	2,420
Mortality	145	19	17	25	206

Source: Disease Control Unit, Ministry of Health and Social Welfare, The Gambia

These measuring variables (morbidity, hospital admission and mortality) for the years 2008, 2009 and 2011 were subjected to statistical test (chi-square) which clearly shows that the observed differences (increases) in these defining variables between the years were indeed statistically significant (p < 0.0001)

The data in Table 3 attest to a long-standing problem, as all the countries except Egypt has more than 10 physicians/10,000 population. Government expenditure on health is less than 2 digits in most African countries while external sources of health financing appears to be huge comparatively to the meagre government spending on health; 2-4 folds higher in 4 of the 6 countries cited. In terms of health infrastructures, radiotherapy unit is < 20/10,000 population in all except South Africa that has a figure of 28/10,000. Table 4 below shows the gender differences in mortality, morbidity and hospital admission.

**Table 3:** Health work force, Finances and Infrastructure for The Gambia and Five (5) other African countries

Country	Physician Workforce (/10,000 population) (2000-2010)	General Government Health Expenditure (as % of Total Government Expenditure)*	External Resources for Health (as % of Total Expenditure on Health)*	Radiotherapy Unit (per 100,000 population) +	Hospital Bed (per 10,000 population)*
Cameroon	1.9	6.1	5.5	0.2	15
Gambia	0.4	11.6	38.0	0	11
Ghana	0.9	8.5	14.0	0.1	9
Egypt	28.3	5.9	0.6	0.7	17
Kenya	1.4	5.8	26.8	<0.05	14
South Africa	7.7	10.4	1.2	0.6	28

Source: World Health Statistics, 2011

+ Note: denotes 2010;

* denotes 2008

**Table 4:** Gender differences in morbidity, hospital admissions and mortality

Gender differences in mortality due to NCDs				
**Year**	**Male**	**Female**	**P -value**	**Overall P -value**
**2011**	114	92	0.03	0.0003
**2010**	77	56	0.01	
**2008**	90	76	0.12	
**Gender differences in hospital admissions due to NCDs**				
**Year**	Male	Female	P-value	<0.0001
**2011**	1145	1275	0.0002	
**2010**	1087	1125	0.2291	
**2008**	997	1206	<0.0001	
**Gender difference in morbidity due to NCDs**				
**Year**	Male	Female	P-value	<0.0001
**2011**	24738	45644	0.0001	
**2010**	24384	43453	0.0001	
**2008**	26846	31921	0.0001	

Globally, there is convincing evidence of gender difference in crude mortality over the years studied with males suffering higher mortality due to NCDs; p-value is 0.0003. However, in the year 2008, there is insufficient evidence of gender difference in mortality due to NCDs.

### Hospital Admission

There is statistically significant evidence gender difference in hospitalisation due to NCDs, p-value is <0.0001. However, in the year 2010, the data suggest insufficient evidence of gender difference in hospitalisation due to NCDs, even though higher number of females were hospitalised due to NCDs.

### Morbidity

There is a numerical difference in the number of people who suffer morbidity due to NCDs with consistently higher number of females than males. These observed gender differences were indeed statistically significant, either by yearly analysis or all the years put together; p-value

## Discussion

### NCDs and Gender

The results of this study suggest statistically significant gender differences in all the defining variables, .i.e. morbidity and mortality as well as hospital admissions. Females are more likely to suffer morbidity than males while men are more likely to die from NCDs than women. Females are more likely to be hospitalised for NCDs than males. These results bring forth the concept of gender inequalities in health which has attracted attentions from diverse disciplines [17–19].

A study in The Gambia shows that there is an association between obesity and female sex, advancing age, non-manual work, urbanity and diastolic hypertension [7]. These findings are not surprising given the socio-cultural context of the Gambia. Many women in the urban environments are housewives, traders or sedentary workers, and are poorly educated with little or no dietary awareness. In many African cultures/societies, being robust or fat is seen as an evidence of affluence or good living or absence of chronic infectious disease such as HIV/AIDS and TB. Hence, this underscores the need for appropriate health education from all disciplines involved in the delivery of health services.

It is not a norm in the Gambia and many other African countries for the female gender to engage in moderate to vigorous physical activities. This may be due to the socialisation process that does not seem to encourage such activities by females. Moreover, previous studies have shown that females are less physically active than males [20–22]. Therefore, the finding in this report is expected; females are more likely to present with morbidity due to NCDs than males, in view of the relationships between obesity, physical inactivity and NCDs.

The proportional difference observed in this study may be explained by the fact that more women seek health care more than men, though men suffer greater mortality than women. In other words, the apparent gender differences noted in morbidity and mortality due to NCDs may be explained by, or is a product of, the synergy between gender differences in health seeking behaviour, health behaviour and lifestyle, socio-cultural influences, and innate biological differences.

### Possible causes of increasing numbers of NCDs in the Gambia

#### Epidemiological and Demographic Transition

The Gambia is in the early phase of nutritional and demographic transition [23] with the resultant effect on chronic NCDs epidemiology. The Gambia, like most developing countries has been experiencing increasing life expectancy [15, 24]. One of the possible reasons for this increasing life expectancy in many African countries is the progress (or stagnation) being made in some areas of the Millennium Development Goals such as reduction in perinatal, infant and maternal mortalities [11, 15]; poverty reduction as seen in the increments in GNI per capita witnessed in the last 2-3 decades; improvement in literacy level, particularly female education, and nutritional disorders, specifically under-nutrition [15]. This resultant longevity (advancing age) is an independent risk factor for most chronic non-communicable diseases [25], and also increases the duration of exposure to risk factors of NCDs. Though, the gains (increasing life expectancy) of the mid 20th century were threatened by HIV/AIDS and Tuberculosis, as many young and middle aged people died of this deadly combo.

There has also been increased investment on health by governments and from external sources, .i.e., international aids and donors [15], even though these funding are sub-optimal to effectively address the double burden of diseases being experienced in Africa. However, this has impacted in some ways (slight decline) particularly in the areas of infectious disease control.

The nutritional transition has been associated with a rapid development of the precursor of many chronic non-communicable diseases, i.e., obesity. Obesity and its associated morbidities (cardiovascular diseases, cancers, arthritis, psychological distress and depression, diabetes and other metabolic disorders) are no longer rare in Africa and other developing countries [1, 4, 11, 26]. In fact, mortality rates due to NCDs have outstripped infectious (communicable) diseases in many African countries, including The Gambia. The emergence of these NCDs is not unexpected as empirical observation shows that an average Gambian diet, particularly among the poor, is starchy (both complex and simple carbohydrate) with excessive oil component, low protein and little vegetable content, predisposing to obesity, and obesity in turn is a risk factor to a number of chronic diseases. This is in addition to the increasing availability of processed (salted), sweetened and fatty food and high calorie drink especially in the urban environments.

### Lifestyle Changes

Non-Communicable diseases and its major ingredient have been referred to, by scholars as diseases of modernity, civilisation or affluences [27, 28]. The quest for modernity or civilisation or better still affluence in Africa and the developing world has been associated with significant economic growth and development in the past 50years. This has impacted positively in improving the GNI per capita, reducing poverty and under-nutrition [15]. Unintentionally, these changes have led to rural-urban migration [15], as young people are more inclined to undertaking service-based (white-collar) jobs deserting agrarian life. Consequently, they become sedentary (physical inactivity) and adopt western lifestyles which are associated with significant risky health behaviours (chronic disease risk factors). Such unhealthy lifestyles and behaviours include smoking and tobacco use, increased and/or excessive alcohol consumption, increased consumption of highly processed fatty, salted food or high energy drinks [2].

Arguably, the upsurge of tourism and trade liberalisation, increased investment and capital flow across the sub-region and globally, and increased access to education and international aids may have been responsible for the increasing urbanity and rural-urban migration in the Gambia and Africa in general. All these elements are entangled within the concepts of globalisation, which is being felt around the world. Furthermore, the globalisation of food and drink marketing as well as trade liberalisation is a major driver of the nutritional transition [10]. These food and drink items are relatively cheap and are low in fibre and micro nutrients [29]. Though not as cheap as obtained in developed world but are becoming more accessible and attractive to both urban and rural inhabitants in Africa.

This globalisation waves are not limited to food and drinks alone, the tobacco companies have moved production facilities from Europe and America to Africa (for growing and production) where governments are more receptive in view of the benefits from tax revenues and employment opportunities for indigenes, thereby inadvertently endangering the life of the populace. These governments and their institutions appear inexperienced, ill-equipped and poorly manned in dealing with the antics of these transnational companies as well as coping with health and economic implications of increased tobacco consumption at the population level. The tobacco companies have also been embarking on innovative and aggressive marketing, targeting the young people [30]. This development is increasing the consumption of tobacco in Africa and many other developing countries, which are a sharp contrast to the declining trends being observed in the developed nations [31]. Even though, there are legal restrictions for the use of tobacco in public places in many African countries, however these laws are not being enforced.

### Any Policy or Control Strategy in Place

#### Structural Level of Intervention

Based on the current trend, there are no concrete policies/programmes put in place in the Gambia, just like many sub-Saharan African countries to stem the emerging epidemic. This is not surprising given the fact that there is little or no reliable data available on chronic non-communicable diseases prevalence and risk factors [11], which is the first step in health policy formulation. Expert such as Unwin and others [32] have recommended three-pronged intervention strategies-epidemiological surveillance, primary prevention (preventing diseases in healthy populations) and secondary prevention (preventing complications and improving quality of life in affected communities). As regards epidemiological surveillance, no recent national survey or even community based survey known has been conducted in The Gambia on the prevalence and/or risk factors for chronic diseases or general health with direct consequences on chronic diseases. However, the health ministry in The Gambia, which is the case in many others African countries, acknowledges the presence and devastating impacts of NCDs [3, 33]. Even though there is a newly formed directorate for NCDs within the ministry of health and social welfare, there has not been yet any significant effort in the areas of NCDs prevention, health promotion, treatment and control.

In response to increasing demands for health services by the population and severe shortage of locally trained manpower, the government established an indigenous university to produce human resources for health, particularly medical doctors, nurses and public health officers. However, the medical training is still evolving and riddled with problems such as dearth of medical teachers, non-availability of specialist trainings in most of the departments and paucity of training facilities. The only teaching hospital in the Gambia still faces challenges of inadequate modern health technologies and related facilities. Furthermore, the existing manpower (mainly foreigners on technical aid programmes) works under pressure and the few local staff are poorly motivated. The existing health personnel to patient ratio are still very low [15], even in the face of the double burden of disease. Generally there is severe shortage of expertise to manage NCDs in The Gambia necessitating referrals to Senegal while the affluent few seek treatment in Europe or the USA.

Overall, there are limited human and infrastructural capacities for non-communicable diseases care and treatment available within The Gambia. This scenario is obtainable in many African countries.

### Community Level of Intervention

The involvements of non-governmental organisations (NGOs) and community-based organisations/groups (CBO) are very minimal in the prevention and care (medical and social support) of chronic non-communicable diseases.

Faith-based organisations (FBOs) are virtually not involved in either the prevention or management of chronic non-communicable diseases in the Gambia. The Pentecostal and Catholic churches play some roles in the prevention of HIV/AIDs and Sickle Cell diseases by encouraging the pre-marital screening for prospective couples who are church members. The Catholic Development Association is actively involved in the care and treatment HIV/AIDS and associated opportunistic infections in The Gambia. Their activities are however not extended to NCDs. The only prominent non-governmental organisation (NGO) that is actively involved in the management of chronic diseases in conjunction with the Royal Victoria Teaching Hospital, Banjul, is Francis De Gaulle Njie Foundation (FDNF). This charitable organisation (FDNF) raises awareness and provides support and care for the affected persons and their families. For example, as part of fund raising programme for the organisation, there was a cancer awareness programme on the 11^th^ of May, 2012, with the theme “Prevention of Common Cancers through Healthy Lifestyles in The Gambia” [34]. There are no organised, community-centred psychos-social and advocacy services for cardiovascular diseases, epilepsy, asthma, sickle cell diseases, chronic liver and renal diseases.

There are a number of fitness centres in The Gambia; some are owned and managed by young men within the community while majority are being managed by private organisations for commercial purposes. Government involvement in this area seems very limited; hence these available facilities are severely under pressure. Most of such centres that are privately owned are well equipped with trained personnel, and these tend to be elitist (exclusive use of the high income earners). Preliminary investigation by the author suggests that most of the young men and women who make use of the community-managed gymnasium and fitness centres do acknowledge the need to improve the facilities available, but this has been hampered by poor financial resources. The vast majority of young adults in The Gambia make use of the fitness centres for cosmetic purposes and to some extent, to keep fit. While they attempt to keep fit and have good body shape, they engage in other behavioural and social risk factors such as smoking, unhealthy diet and to an extent, alcoholism. This underscores the need for appropriate health education (public health messages on risk factors prevention and management of chronic non-communicable diseases) through appropriate channel to the population.

There has been no empirical study done in The Gambia to evaluate the media involvement in chronic diseases prevention and management, however, experience (empirical observation) has shown that media involvement in chronic disease prevention and management has been negligible/non-existent, when compared to diseases targeted by Global Funds (TB, Malaria and HIV/AIDS). Other forms of health communication channels for chronic diseases risk factors are virtually non-existent in The Gambia and in most African countries. However, this is a sharp contrast to that of HIV/AIDS, TB and Malaria. This is a reflection of the fact that public health agenda and implementations in many African countries and developing world in general, are mainly driven by funding and efforts from external sources. Most on-going public health messages are sponsored by donor fund/initiatives which are centred on communicable diseases (particularly, TB, HIV/AIDS and Malaria and Vaccine Preventable Diseases). This calls for urgent responses or local initiatives to combat the growing and devastating epidemic of NCDs in Africa and the developing world.

### Individual Level

The individual level of intervention focuses on the personal behavioural and lifestyle changes that are targeted at preventing the acquisition of the risk factors and also limiting or preventing complication for high risk individuals. This level of intervention also involves pharmacologic interventions that are capable of preventing the onset and or limiting/preventing the potential complications of NCDs. These strategies are at present delivered mainly through the health services which are grossly incapacitated by dearth or non-availability of health workforce and related resources. The level of awareness on the risk factors and management of NCDs seems poor, and public health professionals, private sector, civil society organisations and community groups have to be involved in creating awareness in the areas of prevention, and providing psycho-social support to high risk individuals.

### Limitations of the study

Surveillance and data management for NCDs in the Gambia is still evolving; therefore the NCDs data may be prone to under-reporting or over-reporting. The data are hospital based and may not be a true representation of the morbidity and mortality due to NCDs, as there may be more individuals within the community who do not seek health care due to cost or difficulty with geographical access to health services. This is particularly relevant in the African context where poverty is rife and inverse-care law is very applicable. Apart from this, asymptomatic individuals are more likely to be missed by this hospital-based report, underscoring the need for a population-based survey.

## Conclusion

Chronic NCDs is becoming a serious public health challenge in Africa, and the capacity to address and respond to NCDs in most African countries, particularly, the Gambia is very weak. Hence, African governments should appropriately respond, and be more committed through policy formulation and implementations, taking into account the primary and secondary interventions, consequent upon reliable data gathering processes through efficient surveillance systems.

Experts have advocated the need for multidisciplinary approach to research ranging from cultural, psychological, epidemiological, clinical and economic, as these will inform interventions that will cater for the need of the individual and communities with respect to risk factors and their outcomes [33]. Furthermore, scientific research into molecular basis of NCDs must not be neglected as it will also inform the pharmaco-therapeutics and preventive strategies. All these multidisciplinary research should however translate into interventions that are multi-sectoral and community-based. Africa&apos;s burden of chronic diseases must be addressed given the local context of the risk factors for chronic diseases and human and material resources constraints. Though, there are a few African countries that are heavily endowed with human and material resources which can be effectively channelled to combat the growing epidemic of NCDs, but the sincere political will and selfless leadership appear to be the missing links.

Furthermore, there is paucity of health workforce to cater for the fast growing need for chronic diseases care as well as dearth of medical products and technologies and infrastructural support for efficient and effective service delivery. In fact, evidence suggests decrease in government funding on health with proportionate increase in external health aids, particularly in low income countries [15, 35]. This seems to encourage complacency on the part of the governments (“substitution effects” rather than “complementary effects”) with respect to health care financing. Therefore, there is urgent need to increase investments on human and infrastructural capacity development, and efforts should be directed at preventing brain-drain being experienced in many African countries, by improving working and living conditions for health and allied professionals.

Finally, CBOs, FBOs and NGOs should not leave the control of NCDs in the hands of government alone as they have significant roles to play as seen in infectious diseases like HIV/AIDs and TB.

### Take home messages from the paper

NCDs are becoming a public health challenge in Africa and the capacity to respond, particularly in The Gambia, to halt the progression is weak.

There are increasing cases of mortality, morbidity and hospital admission due to NCDs in The Gambia and there are gender differences in the outcomes; mortality due to NCDs is higher in men while women suffer higher morbidity and hospitalisation.

Periodic epidemiological survey (community-based or national) is warranted to accurately quantify the burden of the NCDs and their risk factors as a first step towards effective policy formulation.

Research into NCDs should be multi-disciplinary in view of their multi-factorial aetiologies and drivers. These multi-disciplinary researches should however translate into interventions that are multi-sectoral, tailored to the local context and community-based.

There is need for better participation of CBOs, FBOs and NGOs in the provision appropriate health education (improving awareness and ways of preventing the risk factors and complication of NCDs), financial and psychosocial supports or management of NCDs.

There is urgent need for increased investments on health workforce and medical products and technologies towards adequately addressing the emerging epidemic of NCDs in The Gambia and Africa in general.

## Additional material

**Annex:** Routine Hospital-Based Data for NCDs in the Gambia
